# The last resort: reducing avoidable employee harm by improving the application of the disciplinary policy and process

**DOI:** 10.3389/fpsyg.2024.1350351

**Published:** 2024-07-19

**Authors:** Andrew Cooper, Kevin Rui-Han Teoh, Ruth Madine, Adrian Neal, Aled Jones, Ammarah Hussain, Doris A. Behrens

**Affiliations:** ^1^Aneurin Bevan University Health Board, Newport, United Kingdom; ^2^School of Nursing and Midwifery (Faculty of Health), University of Plymouth, Plymouth, United Kingdom; ^3^Swansea Centre for Improvement and Innovation, Swansea University, Swansea, United Kingdom; ^4^Birkbeck, University of London, London, United Kingdom; ^5^Department of Clinical Health Psychology Services, North East London NHS Foundation Trust, London, United Kingdom; ^6^Department of Economy and Health, University for Continuing Education, Krems, Austria; ^7^School of Mathematics, Cardiff University, Cardiff, United Kingdom

**Keywords:** disciplinary processes, human resources management (HRM) practices, avoidable employee harm, employee investigations, psychological safety, organizational culture, NHS

## Abstract

**Introduction:**

There is growing evidence within the healthcare sector that employee investigations can harm individuals involved in the process, an organization’s culture and the delivery of its services.

**Methods:**

This paper details an intervention developed by an NHS Wales organization to reduce the number of its employee investigations through an organization-wide focus that promoted a ‘last resort’ approach and introduced the concept of ‘avoidable employee harm’. A range of associated improvement initiatives were developed to support behavior change among those responsible for determining whether an employee investigation should be initiated.

**Results:**

Over a 13-month period, organizational records showed an annual reduction of 71% in investigation cases post-intervention, resulting in an estimated 3,308 sickness days averted annually and total estimated annual savings of £738,133 (based on direct savings and costs averted). This indicates that the organization has started to embrace the “last resort” approach to using employee investigations to address work place issues. The programme was supported with training for those responsible for commissioning and leading the organization’s employee investigations. Analysis of survey data from those who attended training workshops to support the programme indicated that participants showed an increased awareness of the employee investigation process post-workshop and an understanding of the concept of avoidable employee harm.

**Discussion:**

The programme is congruent with the Healthy Healthcare concept, as the study illustrates how its practices and processes have a beneficial impact on staff, as well as potentially on patients. This study highlights wider issues for consideration, including the: (1) the role of Human Resources (HR), (2) taking a multi-disciplinary approach, (3) culture and practice, (4) the responsibility of the wider HR profession.

## Highlights

• The “last resort” approach has been evidenced as an effective way to address the overuse of an organization’s disciplinary policy. However, it will take a lot longer than the initial first thirteen months of the programme to ensure that the new approach is fully embedded.
• The focus on avoidable employee harm supports and drives a change in the application of policies to make them more person-centric.
• For a systemic change in this area and across a sector or profession, there needs to be an acknowledgment of the harm, HR’s contribution to that harm and ownership to lead the change required.
• The study supports the Healthy Healthcare perspective that the practices and processes in one aspect of the organization have an impact on (other) staff and patients, too.
• Emerging research is highlighting problems that are universally inherent with the application of disciplinary policies and processes.

## 1 Background/aims

The Advisory, Conciliation and Arbitration Service describes a disciplinary procedure as “a formal way for an employer to deal with an employee’s unacceptable or improper behavior (‘misconduct’) or performance (‘capability’)” (Advisory, Conciliation and Arbitration Service [ACAS], 2022). In healthcare organizations, disciplinary procedures play an essential role in protecting patients and the public by ensuring healthcare staff maintain professional standards and the delivery of appropriate care (Archibong et al., 2013; Kapur, 2023). However, many organizations are overstretched and under-resourced (Morgan, 2023), balancing concerns around staff wellbeing, retention challenges and the need for more supportive working environments for healthcare workers (Teoh et al., 2023).

There has also been a concern that the disciplinary policy is over-used to address workplace issues (Kline, 2017; Cooper et al., 2024) and that more informal approaches, which are appropriate for many cases, are not being used – leading to a significant impact on organizations and the individuals being taken through disciplinary processes (Hussain, 2022). Against this backdrop, there have been increasing calls for fairer and more compassionate disciplinary processes that are appropriately administered (Kapur, 2023; Neal et al., 2023).

Based on these insights, Aneurin Bevan University Health Board (ABUHB) in NHS Wales commenced work to reduce the number of the investigations it undertook through a commitment to make employee investigations the “last resort.”

This study seeks to test the notion that taking a different approach to the application of the disciplinary policy and process can lead to better outcomes for both employees and their organizations. It does this by drawing on the concept of “avoidable employee harm,” which refers to the potential harm that employees can experience as a result of a workplace factor (like the application of a HR policy), which could be avoided through a more considered implementation of them (Jones et al., 2023). At the same time, it highlights the need for further research to understand the employee’s experience of the investigation process (Morrison et al., 2024) and, to the best of our knowledge, assesses the first intervention to reduce avoidable employee harm.

### 1.1 The impact of employee investigations

The focus on understanding the impact and experiences of individuals being taken through employee investigations, as well as those involved in delivering them and the organization itself is a fairly recent one (Neal et al., 2023; Morrison et al., 2024). At the individual level, being the subject of an investigation can be an emotionally demanding and traumatic process. As a result, staff under investigation have been known to report increased levels of distress, including feelings of anxiety, depression, mistrust and betrayal (Verita, 2018; Hussain, 2022). There are also effects on those involved in delivering or supporting the process, including HR professionals, witnesses called to provide evidence, line managers and trade union representatives (Cooper et al., 2024).

There can also be significant impact at an organizational level. An organization’s culture/s are susceptible to the relational impact of the investigation process. Sensitive details related to the case are often exposed – particularly when the process is flawed or fails to be delivered compassionately (Neal et al., 2023). Problematic perceptions of the investigation process can become a reality and a general view can emerge that the organization does not manage this area of HR practice well. There can also be a significant economic impact on organizations – linked to related periods of sickness or suspension, backfilling roles, internal administration costs and involvement of senior staff (Kline, 2017). And while organizations invest time and resources into unnecessary employee investigations, they are unable to fully deliver on their organizational priorities (Phillips, 2023).

Within healthcare, a potentially significant impact of disciplinary processes can affect the delivery of services to patients. When a frontline member of staff is suspended or takes long-term sick leave it has the potential to impact the quality of care being provided – adding pressure on existing staff (Sizmur and Raleigh, 2018) and where replacement staff are used, leading to issues with continuity of care (Oh and Lee, 2022).

### 1.2 Avoidable employee harm

The recognition that employee investigations can be a source of harm to employees and organizations alike is particularly significant in healthcare, where there is concern around poor staff wellbeing and retention (Kinman et al., 2023). This draws on the concept of “avoidable employee harm,” recently introduced and defined as: “Where harm occurs to employees because of an identifiable and modifiable workplace cause, the future recurrence of which is avoidable by reasonable adaptation, subsequent adherence to and thoughtful implementation of a workplace process or policy” (Jones et al., 2023, p. 60).

Avoidable employee harm parallels the patient safety movement, which identified the avoidability of patient harm within healthcare (Leape, 2021). Whilst it acknowledged the contribution of human error in avoidable harm, it mainly focused on the interaction of system factors (such as processes, lack of time and outcome targets) and human factors (like subjectivity, bias, risk perception and rivalry) within complex systems (Martínez-García and Lemus, 2013).

Over this time, the patient safety movement sought to reduce “avoidable patient harm” through improvement endeavors by recognizing the complexity in which healthcare staff work. Methodologies to support change were developed and a commitment to measurement and continuous improvement to ensure changes made were held and embedded in healthcare systems (Health Foundation, 2021). For example, the implementation of risk assessments and checklists, improved education and training and the simplification and standardization of processes within healthcare have been found to reduce the harm experienced by patients (Wilson et al., 2022; O’Connor et al., 2023).

In most instances, avoidable employee harm results from the failure to adhere or implement correctly a particular workplace process or policy, such as when employees speak up or when experiencing change management and implementation processes (Jones et al., 2023). It draws on the concept of “unintended consequences,” which generally refers to an outcome that has not been anticipated and whilst there can be positive consequences, the focus is usually on the more common negative impacts (Coiera et al., 2016; Behrens et al., 2022; Sommersguter-Reichmann et al., 2023). An example lies in the implementation of health information technologies that are meant to improve efficiency. However, this has also been found to have had the unintended effect of increasing staff workload and increasing errors due to the data entry/retrieval and communication processes involved in using such systems (Ash et al., 2004; Coiera et al., 2016; Kroth et al., 2019). As the definition of “avoidable employee harm” explicitly states that this harm can be mitigated through *reasonable adaptation* (Jones et al., 2023), and it is imperative to assess whether such adaptation actually is possible.

### 1.3 Improving the employee investigation process

Despite the potential for harm from an employee investigation process, it is not feasible to avoid this process given its essential role in ensuring professional standards and the delivery of appropriate care (Kapur, 2023). However, the emerging question lies in how the investigation process can be improved so that standards are held, while at the same time minimizing the potential harm they may bring. While the concept of avoidable employee harm advocates that this can be averted through reasonable adaptation to workplace process or policy (Jones et al., 2023), how this can be done in practice has yet to be tested.

This intersects with an existing gap within the research surrounding the application of employee investigations – not just in healthcare, but more widely across sectors and also within HR theory and practice. Research to date has not focused on interventions to improve the experience of those undergoing the process, but predominately centered on what those experiences might be, including perceptions of justice, psychological harm, bias, and discrimination (Archibong et al., 2019; Bartosiak and Modlinski, 2022; Neal et al., 2023). Where case studies have sought to improve the “effectiveness” of the employee investigation process, this has instead focused on improving performance (Guffey and Helms, 2001) or the consistency of punishments (Shane, 2012).

**FIGURE 1:**
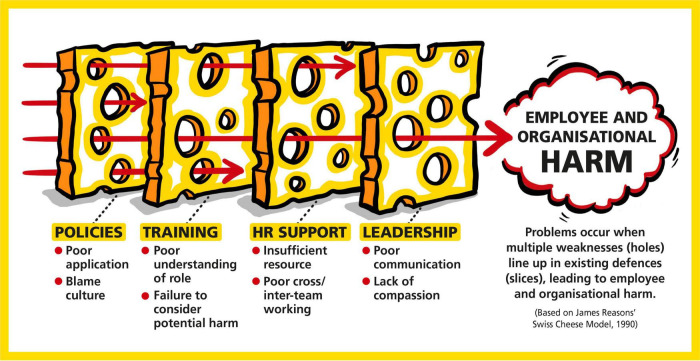
The Swiss Cheese Model and the disciplinary process. The alignment of weaknesses (i.e., the holes) within the layers of defense (i.e., the slices) that results in harm.

Drawing on patient safety theory, the Swiss Cheese Model (Reason, 1990, 2000) of accident causation can be applied to the employee investigation process (Figure 1). The model compares human and system factors to layers of Swiss cheese, with each layer acting as a defense against a potential safety hazard, i.e., something going wrong. In the case of employee disciplinary investigations, there are numerous layers to support the execution of the process. Among these is the policy to guide application, training of staff to ensure policy and guidelines are followed correctly, expertise and support provided by the HR department and compassionate leadership/management styles. However, there are holes in these “layers of defense,” for which the system often develops workarounds or temporary fixes to manage minor failures and mistakes. While breaching one layer may not be critical, the model illustrates how catastrophic events and harms can occur when multiple layers of defenses are breached (when all the holes in the layers of the Swiss cheese line up). Within clinical incidents, the impacts are well documented. However, the model conveys the potentially negative impact on individuals involved in the employee investigation process, when there are failures across it.

### 1.4 The “last resort” approach to employee investigations

Addressing avoidable employee harm in the context of the employee investigation process would require acknowledging and responding to the limitations within the existing system (presented in the Swiss Cheese Model as missing layers of defense or as holes within these layers). In the context of this study, this encompasses a “last resort” approach which seeks to challenge and change the decision-making and behavior of those responsible for commissioning, leading and supporting investigations by (i) helping them recognize the potential harm inflicted on various stakeholders during an investigation; (ii) reducing the overuse of the employee investigation process by exploring alternative options; and (iii) improving the investigation process itself.

In order to deliver this “last resort” approach to employee investigations, it will mean addressing identified barriers from research that currently present as limitations within our Swiss Cheese Model. These include a lack of organizational support (Catley et al., 2017; De Lange et al., 2020); a closed climate (Archibong et al., 2013); time delays (Leonard et al., 2016; Neal et al., 2023); and poor communication (Bennett, 2014; Catley et al., 2017). Conversely, taking a flexible approach with all involved (Jones and Saundry, 2012) and to investigations in general (Neal et al., 2023) could also help mitigate any potential harm on employees.

The factors important to reducing avoidable employee harm are congruent with a compassionate approach, which focuses on relationships by carefully listening, understanding, empathizing and supporting others (Kings Fund, 2022). This allows others to feel valued, respected and cared for, and that are able to meet their potential and carry out their best work. Its importance is reflected in research showing that collective and inclusive leadership in healthcare are associated with better staff wellbeing, safer organizational cultures and patient outcomes (West et al., 2014; West, 2021). However, despite these known barriers and facilitators, to the best of our knowledge there has yet to be an attempt to address these factors and empirically measure its corresponding impact. The various activities of this intervention, based on these theoretical approaches, are described in the Method section below.

### 1.5 Study aims

While the concept and interventions on avoidable harm is well established with patients, the question though is whether this applies in relation to employees. To date, there has not been any attempt to introduce the concept of avoidable employee harm within organizations, nor has there been much research into improving the employee investigation process. This is important from Healthy Healthcare’s emphasis on connecting the healthcare system, staff experience and patient outcomes (De Lange et al., 2020), recognizing that employee investigations can cause avoidable employee harm and adversely impact organizational systems.

Therefore, this study seeks to describe an intervention programme which introduced improvements to reduce the number of employee investigations ABUHB conducted over a thirteen-month period (June 2022 to June 2023) by taking a “last resort” approach. The objectives included:

1. Improving the understanding of avoidable employee harm within the organization.
2. Reducing the number of (avoidable) employee investigations undertaken during the period – thereby removing the related avoidable employee harm.
3. Identifying the number of sickness days averted.
4. Calculating the financial cost of an average investigation and estimating annual financial savings to the organization due to the reduced number of employee investigations commissioned.

## 2 Methods

### 2.1 The organizational setting

Aneurin Bevan University Health Board (ABUHB) is an NHS Wales organization which employs over 16,000 staff. It is responsible for the planning, delivery and commissioning of primary, secondary, community and mental healthcare services for a population of over 660,000 citizens in south-east Wales. ABUHB’s human resources (HR) team had identified, following a review of a 15-month period of employee relations investigations (October 2019 to February 2020), that over 50 per cent of these had led to no sanctions for the individuals who had been taken through them. At the same time, ABUHB’s employee wellbeing service had seen an increase in the number of clients who were experiencing stress and trauma because of undergoing the investigation process.

Within NHS Wales, an all-Wales Disciplinary Policy and Procedure (Welsh Partnership Forum, 2017) has been established as the mechanism for dealing with any disciplinary issues that arise across the service, including within ABUHB.

### 2.2 The intervention programme

**FIGURE 2:**
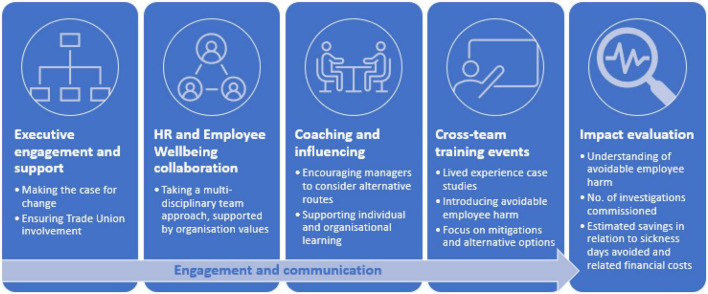
Interventions developed to support the programme.

Recognizing the harm that the disciplinary process can cause was the key driver for developing the organization’s “Improving employee investigations” intervention programme. Crucially, the recognition that over 50% of investigation cases led to no sanction re-enforced the need for a “last resort” approach to be taken. Applying the theories and concepts outlined in this paper, the following activities were undertaken (see Figure 2 for an overview):

(1) Executive engagement and support

Before the programme’s launch, significant work included setting out the case for change and briefing senior and executive leaders. This enabled buy-in, approval and endorsement for the “last resort” approach, which was critical in providing HR and line managers the confidence to follow the new ways being set out. Similar work was also undertaken with trade union representatives to ensure that they understood the course of action and were able to support the approach being proposed.

(2) HR and employee wellbeing collaboration

The programme represented the first time the organization’s HR and employee wellbeing teams had formally worked together. The employee wellbeing service produced an impact assessment of an individual’s experience of the investigation process. It drew on experts from several professional groups (clinical and business psychology, employment law, general practice, quality improvement, HR and leadership development) to better understand the impact on the individuals involved, the organization’s culture, reputation and finances.

(3) Coaching and influencing

The HR team reviewed their practices through the lens of the organization’s values, developing coaching for their members and those responsible for leading and commissioning investigations. The HR team used coaching and influencing skills to encourage managers to identify alternative and informal routes that could be taken, which were included in a framework to support individual and organizational learning. An initial assessment document, used to collect key details, was updated to ensure that as much information as possible was collated to inform decision-making as to whether a formal approach was required. This considered factors such as intent and what previous informal management approaches had been taken to address misconduct and improved learning opportunities.

(4) Training events

The “Employee investigations: Looking after your people and the process” training event was run in July 2022 and February 2023. It included case studies to help attendees understand the impact of the investigation process and an introduction to the new way of working which promoted alternative options to support the “last resort” approach. It was attended by ABUHB’s Executive Director for Workforce and Organizational Development – underlining the organization’s leadership commitment to the direction of travel.

Since the investigation process improvement commenced in June 2022, 128 participants attended one of two workshops. An overview of the available participants is presented in Table 1, indicating that clinicians (50.7%) and those with (no to) little experience overseeing or undertaking investigations (55.2%) were the largest groups of participants. The broader representation was essential to ensure buy-in across organizational functions.

**TABLE 1:** Workshop participant characteristics.

Grouping	*N*
**Total number of participants**	**128 (100%)**
Workshop 1	77 (60.2%)
Workshop 2	51 (39.8%)
**Work groups**	
Clinicians (e.g., nurses, doctors, ward managers)	65 (50.8%)
HR (e.g., workforce business partner, organizational development)	27 (21.1%)
Management (e.g., directors, corporate finance, facilities)	30 (23.4%)
Staff side (e.g., trade union representatives)	6 (4.7%)
**Participants grouped by the number of investigations overseen or undertaken based on surveys completed**	
None	21 (20.0%)
1–5	37 (35.2%)
5–15	17 (16.2%)
15–20	6 (5.7%)
> 20	24 (22.9%)

(5) Engagement and communication

Communication was essential in developing a narrative and presenting a solid case for change that engaged key stakeholders – consistently demonstrating the value of the changes and celebrating those supporting the new behaviors (Cooper et al., 2015). One of the programme’s early adopters was a divisional nurse who works in one of ABUHB’s largest divisions. As a result of the training, they downgraded the number of investigations they were leading, taking a more informal approach instead and completing other investigations in a much shorter timeframe. They had observed the positive and mutual impact on employees and the organization, stating that: “There has been less time spent on investigations, more timely resolution for staff, less stress for staff and a reduction in sickness and absences” (Health Education and Improvement Wales [HEIW], 2023a).

The programme was also supported by producing a video overview, interviews with early adopters and progress updates to key stakeholders – all shared through the organization’s intranet. Leaders of the programme also shared updates and material through their social media channels, which engaged internal audiences and colleagues wider afield. A suite of thought-leadership pieces was developed to maintain and increase engagement with the work (Health Education and Improvement Wales [HEIW], 2023b).

### 2.3 Measures

The following tools were used to collect information to shape the programme’s measures:

(1) Collected through workshop surveys

Three statements were included based on the second level of the Kirkpatrick (1976) evaluation model to assess participants’ learning from the workshops (see Table 2) on knowledge of the investigation process; impact of an investigation on an individual; and how organizations can avoid harm during investigations. This is congruent with the approach that similar studies have sought to evaluate the changes in knowledge pre- and post-training (e.g., Akhanemhe et al., 2021).

**TABLE 2:** Mean rank scores for statements measured pre- and post-workshop.

	Workshop 1			Workshop 2			Total		
**Statement**	**Pre mean rank**	**Post mean rank**	***U***	**Pre mean rank**	**Post mean rank**	***U***	**Pre mean rank**	**Post mean rank**	***U***
I have good knowledge of the processes involved in an investigation	47.35	55.19	*U* = 1072.00	35.20	57.80	*U* = 500.00***	82.90	111.34	*U* = 3182.50***
I have a good understanding of how investigations can impact individuals involved	40.42	63.16	*U* = 697.50***	34.83	58.84	*U* = 466.50***	74.96	121.16	*U* = 2335.50***
I am aware of how organizations can avoid harm to those being investigated as well as those leading investigations	39.94	63.71	*U* = 671.50***	34.96	59.46	*U* = 473.00***	74.30	122.73	*U* = 2267.50***

***p < 0.001.

Each statement was answered on a five-point Likert scale, where 1 = “strongly disagree” and 5 = “strongly agree” and were important to assess any improvement in the understanding of avoidable employee harm within the organization. These three statements were collected through a paper survey administered at the start and end of the workshop.

(2) Collected organizational records

HR records were used to assess the change in the organization’s investigation process, comparing the baseline period (January 2018 to February 2020) and the intervention period (June 2022 to June 2023). The information collected included the number of disciplinaries, the number of decisions taken on whether to pursue the full formal approach or undertake a fast-track process, the outcomes of each disciplinary investigation and start and completion dates (if concluded at the time of collation). Three data metrics were extracted:

• The number of disciplinary cases being commissioned
• The average number of cases per month
• The average number of days taken to conclude an investigation.

### 2.4 Analysis

To meet the first study objective, the mean of the quantitative items from the workshop surveys were compared using a Mann–Whitney-U-test as the data was not normally distributed. For the remaining three objectives we compared organizational data (i.e., number of monthly investigations carried out; number of sick days saved) from the baseline period (January 2018 to February 2020) to the intervention period (June 2022 to June 2023), again using a Mann–Whitney-U test. The cost corresponding financial savings are described in the section below.

## 3 Results

### 3.1 Objective 1: Improving the understanding of avoidable employee harm within the organization

#### 3.1.1 Quantitative pre- and post-workshop comparisons

Comparison of the mean rank scores on items measured pre- and post-workshop (Table 2) showed that overall, there were significant improvements in

• the participants’ knowledge of the process around an investigation (*U* = 3,182.50, *p* < 0.001),
• the participants’ understanding of how investigations can impact the individuals involved in an investigation (*U* = 2,335.50, *p* < 0.001), and
• the participants’ awareness of how to avoid such harm (*U* = 2,267.50, *p* < 0.001).

The same patterns of improvements were observed within each workshop (Table 1), except for no significant differences observed in Workshop 1 on participants’ knowledge of the process around an investigation (*U* = 1,072, *p* > 0.05).

### 3.2 Objective 2: Reduce the number of (avoidable) employee investigations undertaken during the period

#### 3.2.1 Number of cases across the programme

**FIGURE 3:**
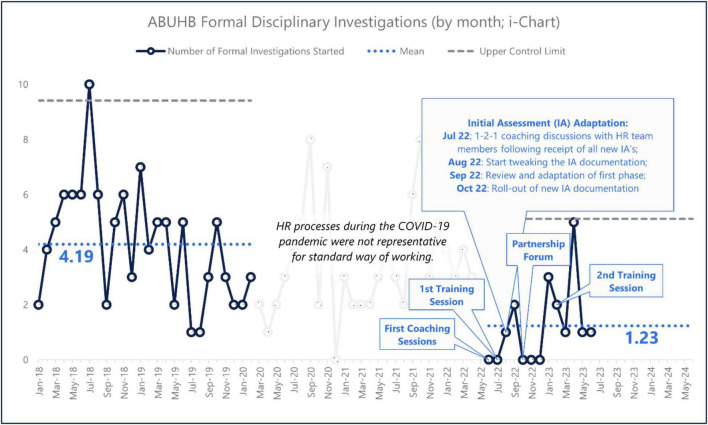
Number of investigation cases per month (January 2018 to June 2023).

Figure 3 charts the monthly fluctuations in the number of cases from January 2018 to June 2023, noting the occurrence of the coaching and workshop sessions. Although available, the period between March 2020 and May 2022 is ignored because it is not representative of standard HR practices, given that this period overlaps with the height of the COVID-19 pandemic.

From Table 3, a statistically significant reduction is seen in the mean number of cases per month from 4.19 cases during the baseline period (January 2018 to February 2020) to an average of 1.23 cases during the intervention period (June 2022 to June 2023) – a reduction of 70.6% per annum, representing a reduction in the related avoidable employee harm (*U* = 38, *p* < 0.001).

**TABLE 3:** Comparison of annual organizational data across the baseline and intervention periods.

	Baseline period (Jan 2018 to Feb 2020)	Intervention period (Jun 2022 to Jun 2023)
Mean number of new disciplinary cases per month	4.19	1.23
Number of new disciplinary cases per year	50 cases	15 cases

### 3.3 Objective 3: Identify the number of sickness days averted due to taking a “last resort” approach to using the disciplinary process.

#### 3.3.1 The average length of an investigation

The average length of a baseline-period investigation (January 2018 to February 2022) was calculated as 265 calendar days. However, the variation in the duration of a baseline-period investigation was substantial (SD = 184). Unsurprisingly, a Mann–Whitney test thus confirmed that the baseline and intervention averages did not *significantly* differ (*p* > 0.01). As a result, the baseline average used for further calculations in relation to estimating sickness days averted and cost savings was 265 days.

In total, an estimated 3,307.5 sickness days were annually averted due to the intervention. This was based on the assumption that 50 per cent of the averted cases (17.5) would have led to sickness absence for the average length of an investigation. HR colleagues derived the 50 per cent figure from the 37 per cent of employees who faced significant distress as a result of being dismissed, resigning or receiving a final warning, which was increased by 13 per cent to incorporate others who would also have found the process significantly distressing, leading to sickness absence.

### 3.4 Objective 4: Calculate the financial cost of an average investigation and estimating annual financial savings to the organization

A cost of £738,133 to the organization was estimated for the 35 additional investigations that would have annually occurred (following the baseline trend) without the intervention. This figure was based on direct annual savings achieved of £586,933 for backfill costs and medical treatment of employees (e.g., GP appointments, medication and mental health support) and annual costs averted of £151,200 for internal administration.

## 4 Discussion

With much of the existing literature focussing on understanding perceptions or barriers to the investigation process (Hussain, 2022; Neal et al., 2023; Cooper et al., 2024), this study goes beyond that by implementing an intervention programme to reduce the avoidable employee harm incurred through the employee investigation process. The “last resort” approach used made a focused attempt to challenge and change the decision-making and behavior of those responsible for commissioning, leading and supporting investigations (primarily senior management, line managers and HR staff) within Aneurin Bevan University Health Board. This was due to the realization that the policy was being over-used and sometimes inappropriately.

Results show that there was not only a better understanding of the concept of avoidable employee harm after the intervention, but a 70.6% reduction in the annual number of investigation cases undertaken. At the organizational level, this was equivalent to an estimated 3,307.5 sick days avoided each year and a saving of £738,133. Therefore, through this intervention we advance the discussion around employee investigations from merely identifying issues to taking proactive steps to address the potential harm to employees and organizations.

### 4.1 Theoretical and conceptual contributions

The study findings provide proof-of-concept through the avoidable employee harm construct that adjustments can be made to extant policies and procedures (Jones et al., 2023). This is vital in testing and refining the scope of what avoidable employee harm is and reinforcing its definition that the underpinning workplace factors are modifiable in nature. It also quantifies that level of harm experienced by the individual employee (i.e., being investigated, going off sick) and the organization (i.e., sick days, financial costs). This is imperative to ensure that avoidable employee harm can build on the avoidable patient harm movement. It further opens the possibility for interventions targeting improved staff experiences on other aspects of organizational policies and procedures, e.g., whistleblowing, change management (Jones et al., 2023).

From a theoretical perspective, we also see the value of the Swiss Cheese Model (Reason, 1990) underpinning the concept of avoidable employee harm within the employee investigation process (Figure 1). The reduction in the number of cases undertaken (and the associated benefits) can be seen as resulting from the reinforcement of organizational defenses against the potential harm from the investigation process to individuals and the organization. The intervention activities help reinforce the need to avoid harm for employees, resulting in a more compassionate approach, better working relationships between stakeholders, and better decision making. All this furthers our understanding of how organizational systems can be strengthened to prevent harm from occurring.

The empirical validation of this proactive approach is congruent with organizational intervention theories that take a preventative approach to manage worker health and wellbeing (Teoh et al., 2023) and adds depth to the theoretical understanding of how interventions can impact the employee investigation process. These results further demonstrate that this programme of work is congruent with the three pillars of Healthy Healthcare, which are worker health, quality of patient care and organizational practice (De Lange et al., 2020), whereby purposeful and overlapping changes at a system level can lead to improvement across the three Healthy Healthcare pillars.

### 4.2 Practical implications and future directions

Reflecting on the advancing future research on both avoidable employee harm and the employee investigation process, the analysis of this work has generated several key insights that should be considered in developing similar work. These can be viewed according to the Swiss Cheese Model (Reason, 1990) where each point reflects a slice of cheese, with the holes being the shortcomings within the slices. Moreover, the Model’s systems approach means that each point should not be viewed in isolation, but collectively as different aspects of the same system. These are:

(1) The role of human resources (HR)
(2) Taking a multi-disciplinary approach
(3) Culture and practice
(4) The responsibility of the wider HR profession

### 4.3 The role of human resources

This study highlights the importance of HR promoting and championing alternative approaches to formal processes wherever possible – which will always be far better for everyone involved. Identifying the problem is only the beginning, and HR needs to lead the change to address issues, sometimes of its own making or deeply embedded beliefs within an organization. This links into best practice guidance around interventions that necessitate the need for the intervention’s stakeholders to appropriately prepare by determining employee and organizational readiness for change, relevant communication strategies and the required leadership support (Leka et al., 2008; Nielsen et al., 2010).

The intervention also demonstrated that HR can take a more active and analytical approach to how they understand and utilize their organizational data, especially in relation to employee investigation processes, including taking responsibility for more systematic analysis and using it to shape necessary remedial action. As with any intervention, there must be an accurate understanding of the issue being focused on so that subsequent activities can be relevant and proportionate to what the data says (Teoh et al., 2023).

### 4.4 Taking a multi-disciplinary approach

The internal partnership between HR and employee wellbeing in ABUHB was a critical element of this work – bringing together very different perspectives and expertise to shape and develop the programme, with recognition from all individuals involved that it would undoubtedly have been less effective without it. The partnership was built on shared values and a common desire to improve the processes and reduce the harm that they were inflicting on employees, epitomizing the systems approach that underpins the avoidable employee harm concept.

Congruent with the research surrounding multidisciplinary (MDT) teams (Taylor et al., 2010; Pillay et al., 2016; Schot et al., 2019), working with other functions provided an opportunity for collaboration, an additional lens, gently challenging a far more comprehensive set of skills and tools to shape the approach to running employee investigations. It also draws on the psychosocial risk management approaches to support staff wellbeing, where multidisciplinary approaches to generate understanding and shared ownership are vital for the long-term success of an intervention (Lavicoli and Di Tecco, 2020). The work to improve employee investigations certainly benefitted from this broader professional expertise and with involvement from occupational health, trade union representatives, quality and safety leads, as well as those focused on developing value-based healthcare. This directly links to the Healthy Healthcare concept, which advocates for cross-collaboration of units across a system to enhance a better understanding of issues as well as improving solution development and implementation (Løvseth and Teoh, 2023).

### 4.5 Culture and practice

The “last resort” approach recognizes that there is also an underlying issue of how the organization responded to employee incidents – not the intentional, malicious and criminal error that fully merited a formal investigation, but instead lower-level incidents that happened as the result of recognized human error and working in a complex environment.

A similar approach has been taken within Mersey Care NHS Foundation Trust, mainly through a restorative just culture approach, which aimed “to replace hurt by healing in the understanding that the perpetrators of pain are also victims of the incident themselves” (Kaur et al., 2019). The Mersey Care NHS Foundation Trust had seen a similar impact with a reduction in suspensions and dismissals, an increase in the reporting of adverse events, as well as a reduction in staff absence and improved retention (Social Partnership Forum, 2020). Within ABUHB, the work to improve employee investigations was aligned with its organizational values – facilitating the engagement and support of relevant stakeholders – including senior leaders from corporate and clinical functions. From a sustainability perspective, it meant that the activities could be aligned with existing organizational priorities, making it easier to gain resourcing and support.

In terms of Healthy Healthcare, culture and practice influence the perception of good practice and values, norms and unwritten rules that are reflected in daily work activity, behavior, perspectives, attitudes, and work environment (Løvseth and Teoh, 2023). Therefore, culture and practice has an impact on all three Healthy Healthcare pillars: healthcare worker health and wellbeing, organizational practice and quality of care.

### 4.6 The responsibility of the wider HR profession

There is increasing scrutiny of the employee investigation process on a wider level – which highlights, at best, the process’s ineffectiveness in addressing issues and, at worst, its capacity to make situations infinitely worse (Hussain, 2022). Within NHS England, Kline argued that “[…] disciplinary policies, procedures and training intended to set standards, which (in theory) emphasize learning but in practice may prompt blame, prolong processes, be adversarial and, like referrals to professional regulators, be prone to bias against BME staff” (Kline, 2023, p. 315).

Internationally, a review undertaken in Norway highlights the structural challenges and approach of the process, where the default position is to take an inquisitorial position as the employer not only determines the rules and regulations but also takes on the role of prosecutor, police and judge, all at the same time (Kuldova and Nordrik, 2023). This showcases where power is situated within the wider system, undermining the need for participation, control, and the voice of the employee within the workplace. Similarly, a case study on the law that regulates disciplinary processes in the Brazilian Federal Public Service concluded that the disciplinary perspective is akin to institutional corruption that allows for administrative abuse (Andrews, 2023). Evident across both examples is how the system has the responsibility for better investigation processes as a way to reduce the prospect of harm to the workforce (Jones et al., 2023).

A handful of individual programmes, as highlighted in this paper, is barely scratching the surface of a more ingrained problem that needs to be owned by HR’s professional bodies. It is only when these bodies acknowledge the significant harm of these processes and own it as an issue that they are best placed to resolve, that we will see the level of change needed to make the process safer and fairer for those being taken through it and those charged with running it. At the same time, the leaders of organizations need to recognize the issue as not solely a problem for HR policy and application, but one that threatens the wellbeing of their organizations and that addressing these issues has the potential to bring about significant improvements in wellbeing at a system level (Teoh et al., 2023). The damage that organizational practices, like employee investigations, inflict demonstrates how this one pillar affects both employees and patients (De Lange et al., 2020).

### 4.7 Limitations

The study’s implications need to be framed within several limitations. First, the intervention period immediately followed the end of the COVID-19 pandemic. During the pandemic, the practices around disciplinaries were not “normal,” potentially affecting the robustness of data collection between March 2020 and May 2022. Consequently, this period was excluded from the analysis. Second, the measures used to assess knowledge learning through the workshop were not established validated measures. Although they were developed specifically for this study, they draw on the Kirkpatrick (1976) model of evaluation and were reviewed for face validity. Third, improvement was diagnosed by using a process control chart, not testing for a structural break of the time series due to the relatively short length of the time series (after excluding COVID-19).

Fourth, the financial calculations are a conservative estimate and do not include any legal and court-related costs. As Kline sets out, “the total cost of unnecessary disciplinary investigations, suspensions, hearings, and appeals for all staff groups […] is many times higher today, especially when supplemented by unnecessary referrals to professional regulators and the additional cost of related sickness absence, staff cover, early retirement and turnover” (Kline, 2017).

Finally, we do not capture the experience of employees themselves undergoing an investigation as the evaluation focused on measuring outcomes and improvements on reducing the number of cases as a representation of the reduced harm. Nevertheless, the congruence of the findings across the different data sources indicating a positive improvement in learning from the workshops with the organizational records showing reduced employee harm in relation to cases, costs, and sickness absence, provides cross-validation for the findings observed and reinforce the study’s conclusions.

## Data availability statement

The raw data supporting the conclusions of this article will be made available by the authors, without undue reservation.

## Ethics statement

Ethical approval was not required for the studies involving humans because Work undertaken was undertaken as service evaluation and our formal approval process (which was sought and gained) constitutes “Local Governance” with peer review (confirmed 19.07.23). The studies were conducted in accordance with the local legislation and institutional requirements. Written informed consent for participation was not required from the participants or the participants’ legal guardians/next of kin in accordance with the national legislation and institutional requirements because Work undertaken was undertaken as service evaluation and our formal approval process (which was sought and gained) constitutes “Local Governance” with peer review (confirmed 19.07.23).

## Author contributions

AC: Conceptualization, Formal analysis, Funding acquisition, Investigation, Methodology, Project administration, Resources, Supervision, Writing – original draft, Writing – review & editing. KT: Conceptualization, Data curation, Formal analysis, Methodology, Writing – original draft, Writing – review & editing. RM: Data curation, Formal analysis, Investigation, Project administration, Resources, Visualization, Writing – review & editing. AN: Conceptualization, Formal analysis, Funding acquisition, Investigation, Supervision, Writing – review & editing. AJ: Conceptualization, Formal analysis, Writing – review & editing. AH: Writing – review & editing. DB: Conceptualization, Data curation, Formal analysis, Investigation, Methodology, Visualization, Writing – review & editing.
